# IgG4-related disease as a rare cause of gastric outlet obstruction: a case report and literature review

**DOI:** 10.1186/s12876-021-01927-x

**Published:** 2021-09-20

**Authors:** Lina Chen, Abdulaziz Almudaires, May Alzahrani, Karim Qumosani, Subrata Chakrabarti

**Affiliations:** 1 Department of Laboratory Medicine and Molecular Diagnostics, Sunnybrook Health Sciences Centre, University of Toronto, Toronto, ON Canada; 2 Department of Pathology and Laboratory Medicine, London Health Sciences Centre and Western University, London, ON Canada; 3 Division of Gastroenterology, Department of Medicine, London Health Sciences Centre and Western University, London, ON Canada

**Keywords:** IgG4-related disease, Gastric outlet obstruction, Gastrointestinal ulceration, Case report

## Abstract

**Background:**

IgG4-related disease involvement of the digestive tract is very rare. In few reported cases of isolated gastric/duodenal IgG4-related disease, none of which resulted in luminal obstruction.

**Case presentation:**

A 59 years old female presented with longstanding gastrointestinal symptoms. CT showed mural thickening of the proximal duodenum. Gastroscopy showed antral ulcer extending into the duodenum with outlet obstruction and biopsy showed acute on chronic duodenitis. Whipple’s procedure was performed and IgG4-related disease was diagnosed on final pathology. Symptoms were revolved on mycophenolate mofetil and prednisone with no recurrence.

**Conclusions:**

Our case is the only reported case with gastric outlet obstruction secondary to gastroduodenal IgG4-related disease. The diagnosis should be considered in the differential diagnosis of unexplained duodenal stricture, gastric outlet obstruction or gastrointestinal ulceration. IgG4-related disease usually responds to steroids but long-term response rates to steroid-sparing agents, especially in the subset of patients with luminal IgG4-related disease remains to be determined.

## Background

IgG4-related disease (IgG4-RD) is a systemic inflammatory disorder characterized by abundant infiltration of IgG4-positive plasma cells in the affected organs [1]. The liver, biliary system and pancreas are the most commonly affected organs [2, 3]. The most common form of undiagnosed IgG4-RD leading to a Whipple resection is type 1 autoimmune pancreatitis, which may present as a mass lesion mimicking pancreatic ductal carcinoma clinically [2, 4, 5]. IgG4-RD involvement of the digestive tract is very rare [6, 7]. To date, only a few cases of isolated gastric/duodenal IgG4-RD have been reported, none of which resulted in luminal obstruction [8–13]. Because IgG4-RD can mimic various disorders, correlation between clinical, pathological and radiological findings is often required to establish the diagnosis [14]. In terms of treatment, immunosuppression remains the cornerstone of management, especially in symptomatic patients, with surgery reserved for highly fibrotic lesions unresponsive to medical therapy [14, 15].

## Case presentation

**Fig. 1:**
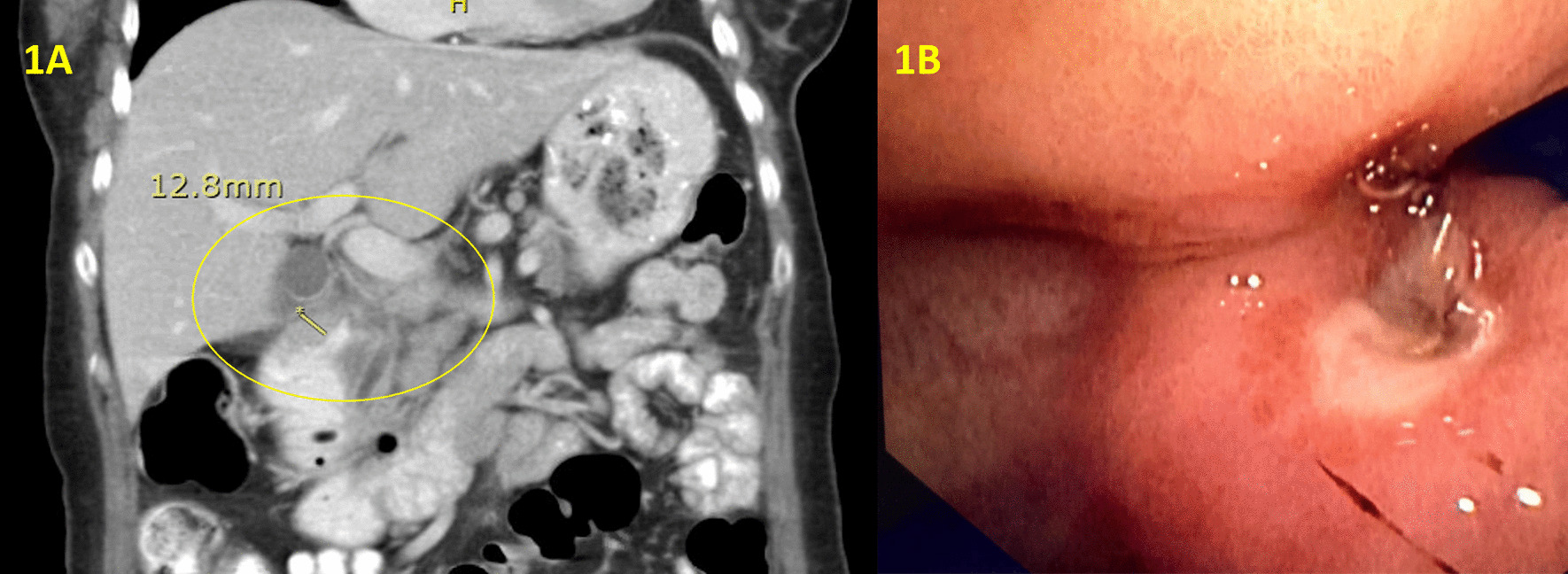
Preoperative CT showed mural thickening of the proximal duodenum (**A**). Gastroscopy showed a clean based antral ulcer extending into the duodenum with severe obstructing duodenal stricture (**B**)

A 59 year old female with a past medical history of Guillain-Barré syndrome and previous gastric ulcer on multiple medications including naproxen 500 mg and omeprazole 20 mg daily presented with longstanding abdominal pain and progressive postprandial nausea, vomiting and weight loss over 6 months’ period. CT showed mural thickening of the proximal duodenum (Fig. 1A). The patient received gastroscopy on multiple occasions, the most recent gastroscopy showed a clean-based antral ulcer extending into the duodenum with severe obstructing duodenal stricture that impeded the ability to pass an adult size gastroscope (Fig. 1B). Biopsy of the duodenal stricture showed acute on chronic duodenitis with gastric biopsies negative for helicobacter pylori infection. Initially, peptic ulcer disease was thought to be the etiology. However, malignancy could not be ruled out, and surgical management with Whipple’s procedure was therefore pursued.

**Fig. 2:**
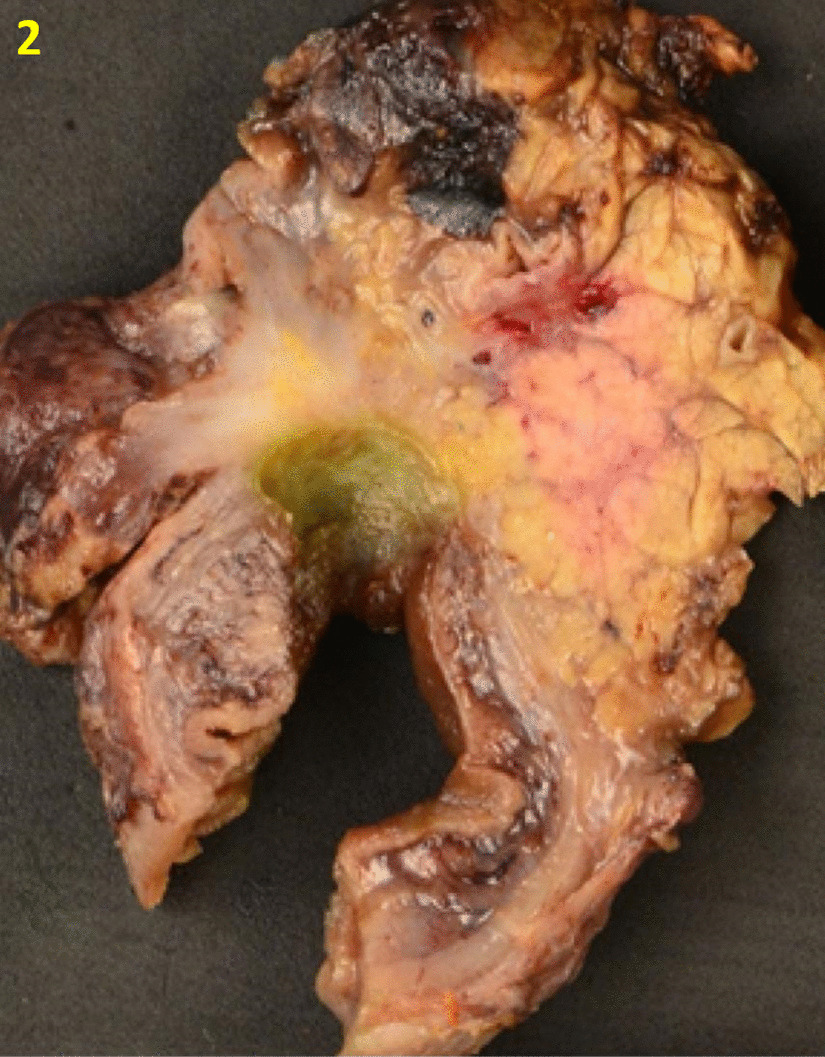
Gross examination of the resected Whipple specimen revealed a large ulcerated mass involving stomach and duodenal wall

**Fig. 3:**
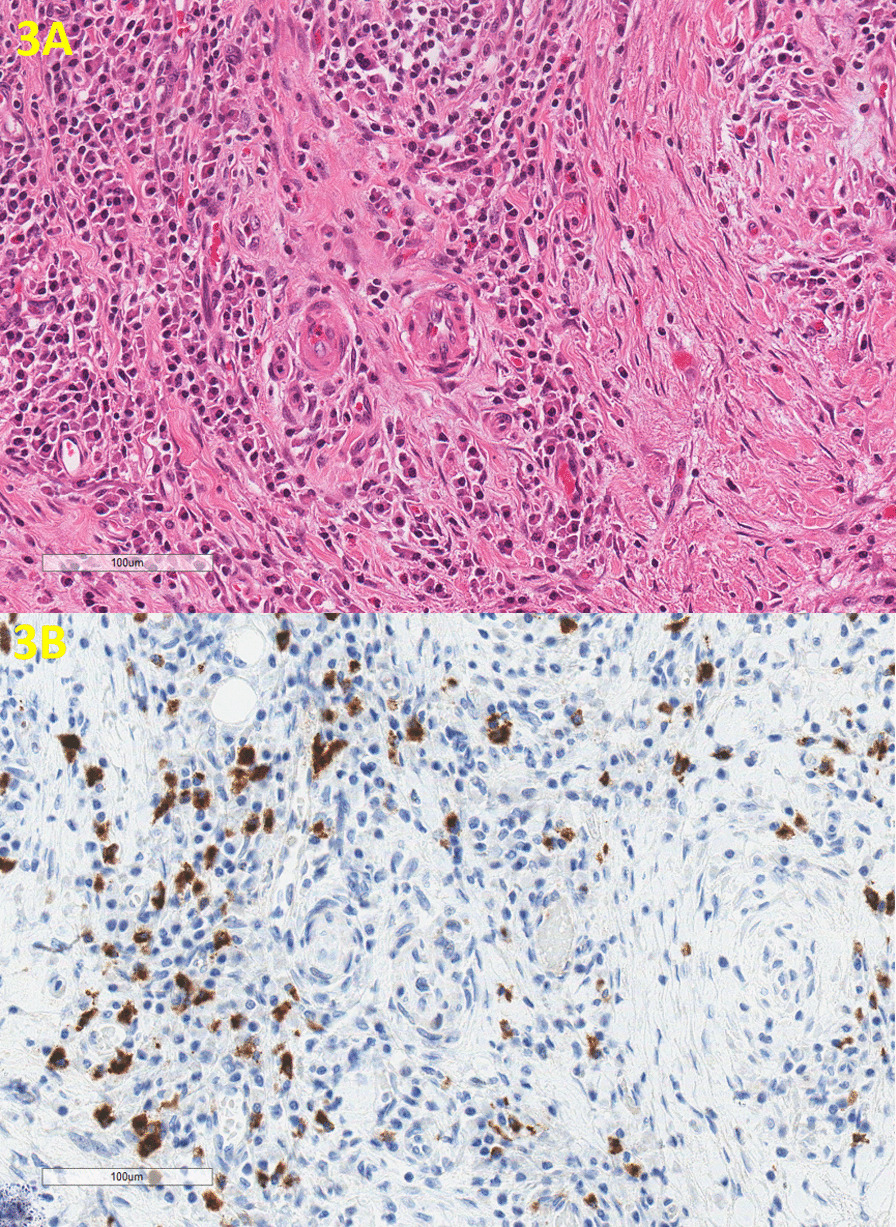
There is extensive sclerosing fibrosis and lymphoplasmacytic inflammation (3A, H&E, 200x) with abundant IgG4 positive plasma cells (3B, IgG4 stain, 200x)

Gross examination of the resected specimen revealed a large ulcerated mass (2.5 × 1.2 × 1.0 cm) involving stomach and duodenal wall (Fig. 2). Microscopically, sections showed a duodenal ulcer and associated underlying extensive sclerosing fibrosis and lymphoplasmacytic inflammation involving the duodenal wall and extending into the adjacent pancreas (Fig. 3A). There was no malignancy or dysplasia present. IgG4 stain demonstrates up to 50 IgG4 positive cells per high power field (Fig. 3B).

Postoperatively, the patient developed an anastomotic leak that was managed conservatively with antibiotics and drainage resulting in significant symptomatic improvement. MRCP did not show any pancreatic or biliary abnormalities.

**Fig. 4:**
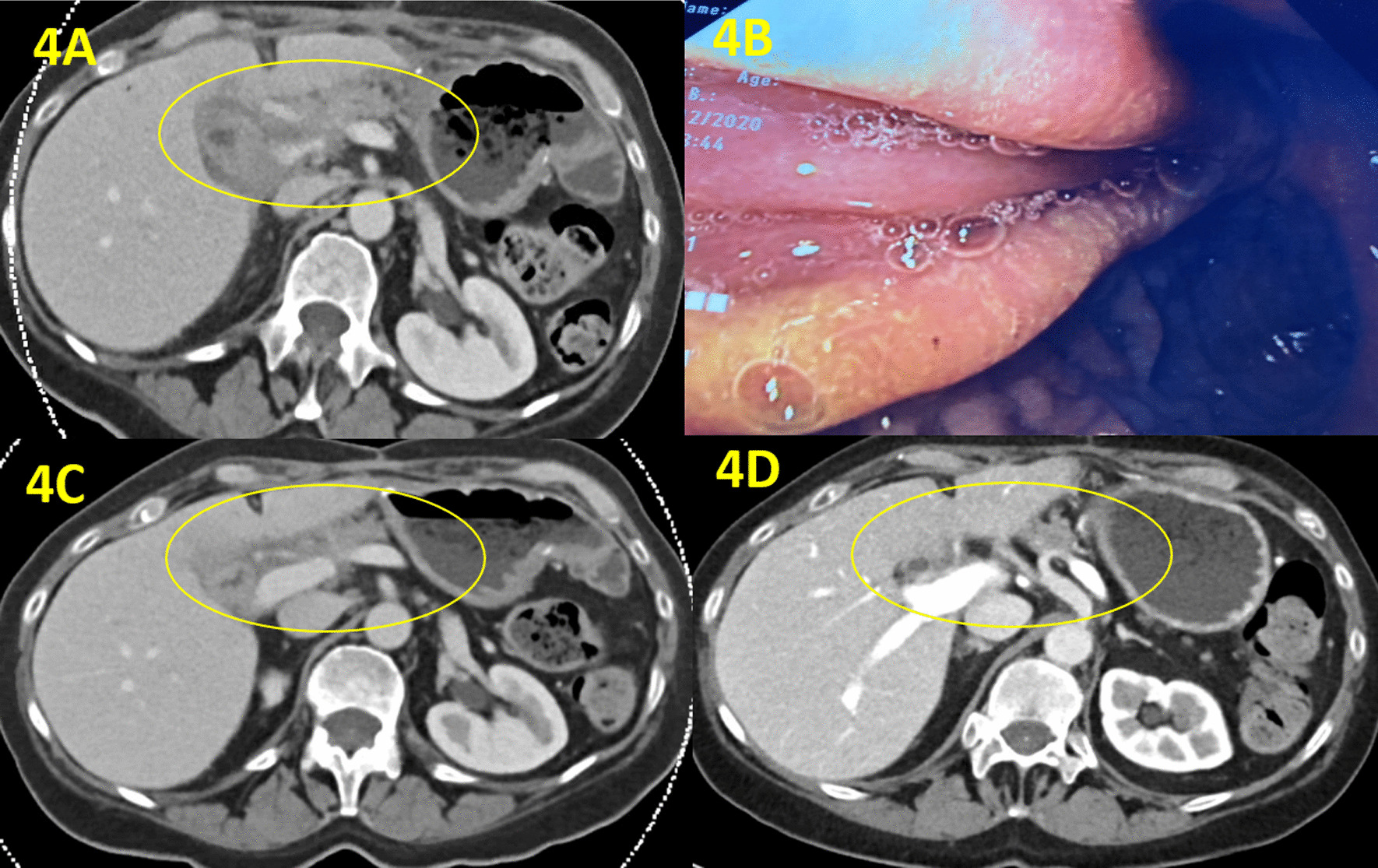
Postoperatively, CT scan revealed inflammatory changes at the anastomosis site with severe duodenal thickening, representing recurrent IgG4-RD (**A**). Upper endoscopy showed very subtle perianastomotic erosions and erythema (**B**). Two weeks after prednisone therapy, follow-up CT showed marked improvement of the perianastomotic changes (**C**). Another follow-up CT at 3 months after starting mycophenolate mofetil therapy revealed no recurrent disease (**D**)

A few weeks later, she represented with abdominal pain, nausea and vomiting, with trivially elevated serum IgG4 at 0.965 g/L (0.039–0.864 g/L). CT scan revealed inflammatory changes at the anastomosis site with severe duodenal thickening (Fig. 4A). Therefore, upper endoscopy was repeated; which showed very subtle perianastomotic erosions and erythema (Fig. 4B), and a patent small bowel with non-diagnostic random biopsies. These changes likely represented recurrent IgG4-RD. To induce remission, prednisone was started (1 mg/kg, then 40 mg), with symptom resolution within two weeks, and marked improvement of the perianastomotic changes on follow up imaging (Fig. 4C). Subsequently, she was started on mycophenolate mofetil 500 mg with a slow prednisone taper, with no recurrence at 3 months (Fig. 4D).

Retrospectively, the patient’s pre-operative duodenal biopsy was reviewed and IgG4 stain was performed. In addition to the chronic and active duodenitis reported initially, there was ulceration with submucosal fibrosis and inflammation. IgG4 positive cell counts were 27/HPF.

## Discussion and conclusions

IgG4-RD is an uncommon inflammatory condition of the GI tract that can potentially cause chronic gastric ulceration, mainly when marked fibrosis along with lymphoplasmacytic infiltrates and lymphoid follicles are present within the ulcer and adjacent tissue.

Untreated IgG4-RD can lead to significant morbidity, organ dysfunction and sometimes death [16]. However, the natural history and long-term prognosis of IgG4-RD is not yet well understood. Active IgG4-RD responds very well to glucocorticoids, and their use as a first line agent for induction of remission is recommended [17]. A common initial treatment is prednisone at 30–40 mg per day for 2–4 weeks, followed by a slow tapering schedule, with the aim of discontinuing steroids in 3–6 month after initiation. However, because relapse is not uncommon with glucocorticoid tapering and discontinuation, the use of maintenance therapy early in the course is often needed [16]. This can be in the form of a conventional steroid sparing agent (e.g. mycophenolate mofetil or azathioprine), Rituximab, or low dose steroids (prednisone 2.5–5 mg/day), though the latter poses a higher risk for long-term toxicities. Long standing, highly fibrotic disease (e.g. fibrotic orbital pseudotumors and sclerosing mesenteritis), response to medical treatment can be poor and surgical resection can be a better option [15, 16]. Another indication for surgery is the inability to obtain diagnostic tissue and rule out malignancy with less invasive techniques [15, 16].

Serum IgG4 levels were initially thought to be a key diagnostic feature of IgG4-RD, but recent evidence has de-emphasized the value of elevated serum IgG4 levels [18]. The key to diagnosis is immunohistochemical demonstration of tissue infiltration by IgG4-bearing plasma cells with morphological evidence of lymphoplasmacytic infiltrates, storiform fibrosis, and obliterative phlebitis [2]. Higher serum IgG4 levels might be helpful in diagnosis of IgG4-RD, however, a low positive predictive value has been reported [18]. Many other conditions can lead to serum IgG4 elevation. In addition, a completely normal level of IgG4 does not exclude a diagnosis of related disease, although a higher level increases the diagnostic yield [2, 18].

IgG4 disease-like features can be found adjacent to malignant tumors [4]. Therefore, a biopsy diagnosis of IgG4 related disease cannot rule out malignancy entirely. However, in cases presenting with mass lesions albeit with biopsy results showing increased IgG4 plasma cells and fibrosis, present a clinical dilemma. The question remains if one should treat it as IgG4-related disease or repeat biopsy or proceed with surgery. The pathogenesis of IgG4-related disease is still vague and immature. There are two prevailing theories underlying observed pathology [3]. One is the induction of a polarized CD4 + T cell that activates innate immune cells responsible for the development of fibrosis. The other hypothesis is a negative feedback process involving generation of IgG4 secreting plasmablasts, plasma cells, and IgG4 antibodies to prevent autoimmune response. Autoimmunity has been hypothesized to be a potential initial immunologic stimulus for the Th2-cell response in IgG4 related disease. Therefore, in patients with autoimmune conditions presented with biopsy negative mass lesion, IgG4 -related disease should always be included in the differential diagnosis.

Our case presented with gastric outlet obstruction, an extremely rare complication of IgG4-RD. To our knowledge, there are 10 reported case in the literature to present with gastrointestinal luminal obstruction. Among the 10 cases yielded in our literature search, intestinal obstruction was secondary to sclerosing mesenteritis in 3 cases [19–21]. Obstruction secondary to intestinal IgG4- RD was reported 5 times in the literature; 1 involving the colon, 2 in the small bowel, and 2 involving both the large and small intestine, including the ileocecal valve, one of which involved the appendix as well [22–26]. Among these 10 cases, there is only one case of gastric outlet obstruction as a complication of IgG4-RD. However, this was secondary to pancreatitis [27], not from direct involvement of stomach. Therefore, our case is the first one to report as isolated gastric/duodenal IgG4-RD resulted in gastric outlet obstruction.

Serum IgG4 level was elevated in 4 case reports [25–27]. Similar to our case, majority of the reported cases required surgery to reach the diagnosis. However, EUS yielded the diagnosis in 2 cases [21, 27]. Only half of the patients received steroids and 2 patients receiving additional immunosuppressants postoperatively [19–28].

Another interesting finding in this case is that our patient has a past history of Guillain–Barre syndrome. Studies have shown that the presence of IgG4 antibodies are extremely specific for diagnosis of chronic inflammatory demyelinating polyneuropathy (CIDP) which is considered the chronic counterpart of Guillain–Barre syndrome [29–31]. This raises the possibility that the patient may have long standing IgG4 related disease before presenting with the gastroduodenal obstruction.

Additionally, preoperatively, the patient was discovered to have iron deficiency anemia. Colonoscopy was also performed which revealed patchy erythematous changes across the right colon up to the proximal transverse colon, with colonic biopsies showing chronic colitis with focal activity and ulceration. A diagnosis of inflammatory bowel disease (IBD) was suspected. However, following the Whipple resection and the subsequent diagnosis of IgG4-RD, we retrospectively performed IgG4 stain on the colonoscopic biopsy specimen which showed up to 21 IgG4 positive plasm cells per high power field. On one hand, given the context, colitis secondary to IgG4-RD is very plausible. On the other hand, IgG4-positive plasma cells are commonly seen in IBD, especially ulcerative colitis [32, 33]. Therefore, the etiology and significance of the inflammation seen on colonoscopy remains to be determined. Of note, our patient describes no diarrhea, change in bowel habits or extra intestinal manifestations of IBD.

IgG-RD should be included in the differential diagnosis for unexplained duodenal stricture, gastric outlet obstruction and/or gastrointestinal ulceration. In order to diagnose or exclude IgG4-RD, a thorough assessment and correlation between the clinical and histopathological findings is crucial. Due to the disease’s insidious and multi-organ nature, obtaining a detailed history of the symptoms, organ involvement, chronicity and disease evolution, supported by radiological examination of the abdomen and the common organs affected by IgG4-RD is very helpful; but often not satisfactory to exclude the diagnosis. Because of its mimicry of many conditions, it is very difficult to rule out IgG4-RD without histopathological assessment with IgG4 staining. In gastrointestinal disease, where endoscopic biopsies are inconclusive or fail to rule out IgG4-RD, obtaining biopsies from another affected organ (e.g. Endoscopic ultrasound guided biopsies from the pancreas or lymph nodes) can be sought. Moreover, surgical resection should be the final resort to establish a tissue diagnosis, especially if malignancy has not been excluded.

In conclusion, IgG4-RD involving the gastrointestinal tract is rare with cases reported in the literature presenting in variable ways. Our case is the only reported case with gastric outlet obstruction secondary to gastroduodenal IgG4-RD. This unique fibroinflammatory condition should be considered in the differential diagnosis of unexplained duodenal stricture, gastric outlet obstruction or gastrointestinal ulceration. IgG4-RD usually responds to steroids but long-term response rates to steroid-sparing agents, especially in the subset of patients with luminal IgG4-RD, remains to be determined.

## Data Availability

Not applicable.

## Abbreviations

IgG4-RD: IgG4-related disease
CIDP: Chronic inflammatory demyelinating polyneuropathy
IBD: Inflammatory bowel disease
